# Effects of Subtoxic Concentrations of Atrazine, Cypermethrin, and Vinclozolin on microRNA-Mediated PI3K/Akt/mTOR Signaling in SH-SY5Y Cells

**DOI:** 10.3390/ijms232314538

**Published:** 2022-11-22

**Authors:** Agnese Graziosi, Giulia Sita, Camilla Corrieri, Sabrina Angelini, Roberta d’Emmanuele di Villa Bianca, Emma Mitidieri, Raffaella Sorrentino, Patrizia Hrelia, Fabiana Morroni

**Affiliations:** 1 Department of Pharmacy and BioTechnology—FaBiT, Alma Mater Studiorum—University of Bologna, via Irnerio 48, 40126 Bologna, Italy; 2 Department of Pharmacy, School of Medicine and Surgery, University of Study of Naples—Federico II, via Montesano 49, 80131 Naples, Italy; 3 Department of Molecular Medicine and Medical Biotechnology, School of Medicine and Surgery, University of Study of Naples—Federico II, via Pansini 5, 80131 Naples, Italy

**Keywords:** atrazine, cypermethrin, vinclozolin, endocrine-disrupting chemicals, neurotoxicity, microRNA

## Abstract

Endocrine-disrupting chemicals (EDCs) are different natural and synthetic chemicals that may interfere with several mechanisms of the endocrine system producing adverse developmental, metabolic, reproductive, and neurological effects in both human beings and wildlife. Among pesticides, numerous chemicals have been identified as EDCs. MicroRNAs (miRNAs) can regulate gene expression, making fine adjustments in mRNA abundance and regulating proteostasis. We hypothesized that exposure to low doses of atrazine, cypermethrin, and vinclozolin may lead to effects on miRNA expression in SH-SY5Y cells. In particular, the exposure of SH-SY5Y cells to subtoxic concentrations of vinclozolin is able to downregulate miR-29b-3p expression leading to the increase in the related gene expression of ADAM12 and CDK6, which may promote a pro-oncogenic response through the activation of the PI3K/Akt/mTOR pathway and counteracting p53 activity. A better understanding of the molecular mechanisms of EDCs could provide important insight into their role in human disease.

## 1. Introduction

**Figure 1:**
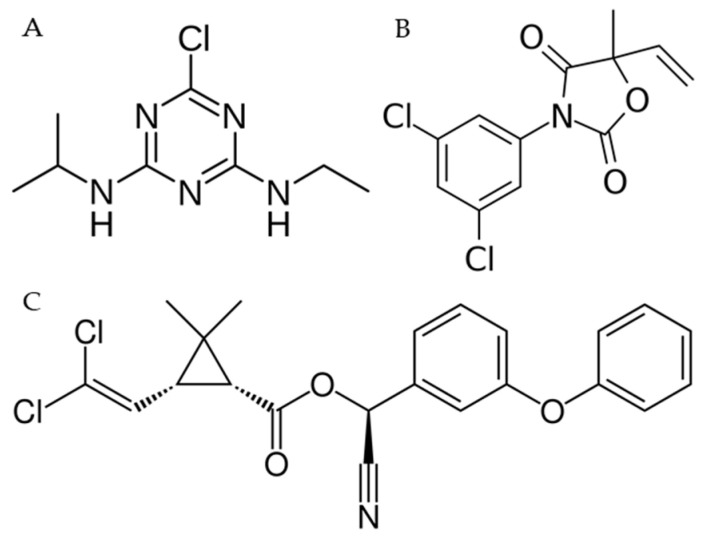
Chemical structures of ATZ (**A**), VNZ (**B**) and CYP (**C**).

Endocrine-disrupting chemicals (EDCs) represent a worldwide issue for environmental and human health [1]. EDCs deriving from agricultural and industrial sources, such as pesticides, herbicides, and chemicals used in the plastics industry and in consumer products, are generally found in the environment [2]. EDCs have received increasing attention for their ability to alter the endocrine and homeostatic systems by interfering with endogenous hormones from synthesis to elimination, through transport, metabolism, and receptor binding [3]. Pesticides are designed to be active against specific targets, but they may cause toxicity to other nontarget species by different modes of action counting the interference with the endocrine function. Indeed, pesticides have been found to alter the function of hormone receptors by binding, to interfere with the synthesis or clearance of endogenous hormones, and to interact with neurotransmitter systems, causing yet other effects with still poorly understood mechanisms [4]. The category of pesticides includes herbicides used to destroy weeds and unwanted vegetation, insecticides for limiting the diffusion of a wide variety of insects, fungicides useful to antagonize the growth of molds and mildew, disinfectants for avoiding the uncontrolled spread of bacteria, and compounds used to keep off mice and rats. Because of the extensive use of chemicals in agricultural production, people are exposed to regulated low levels of pesticide residues through their diets. Atrazine (ATZ, 6-chloro-4-N-ethyl-2-N-propan-2-yl-1,3,5-triazine-2,4-diamine, Figure 1A) is one of the most commonly diffused herbicides in surface and groundwater, where it persists, as its half-life is around 95–350 days and it is hardly degraded [5]. ATZ is banned in Europe, but it is the second most commonly used herbicide besides glyphosate [5]. Recent studies have shown in various species, including fish, amphibians, reptiles, and mammals, the reduction and induction in androgen and estrogen levels, respectively, which represent a possible mechanism able to explain the effect of ATZ on male development [6,7]. Cypermethrin (CYP, [cyano-(3-phenoxyphenyl)methyl] 3-(2,2-dichloroethenyl)-2,2-dimethylcyclopropane-1-carboxylate, Figure 1B), a pyrethroid pesticide, is largely used in agricultural production and daily life. CYP is stable to hydrolysis with a half-life of greater than 50 days [8]. Recently, several studies have demonstrated that CYP could dramatically influence male reproduction and fertility because of its anti-androgenic properties [9]. Vinclozolin (VNZ, 3-(3,5-dichlorophenyl)-5-methyl-5-vinyl-1,3-oxazolidine-2,4-dione, Figure 1C) is a dicarboximide fungicide used in several crops such as oilseed, vines, fruits, vegetables, ornamental plants, and turf where it inhibits spore germination. In terrestrial field dissipation studies, VNZ has an half-life of 34 to 94 days and its residues (vinclozolin plus its dichloroaniline-containing metabolites) were 179 to >1000 days [10]. VNZ is considered an EDC pesticide and both itself and its metabolites show antiandrogenic effects blocking the androgen binding site of the receptors [11,12].

Much effort has been spent to investigate the impact of EDCs on the reproductive system. Recent data highlight that exposure to EDCs may produce abnormalities in behavior and brain functions [13]. Thus, it can be hypothesized that exposure to these toxic compounds could modify specific molecular mechanisms or signaling pathways, causing alterations in the structure and function of the central nervous system (CNS). Indeed, the consequences of EDC exposure on early development were deeply investigated, but so far, the effects on the adult and aging brain are mostly unknown. Gonadal hormones are fundamental for the CNS’ activity during maturity and even senescence not only for their role in the neurogenesis processes, but also because they can exert a protective activity counteracting the insurgence of neurodegenerative disorders [14]. Moreover, EDC exposure may impact neurotransmitters also outside of the hypothalamus, which is known as a neuroendocrine structure [15]. Exposure to EDCs, as atrazine, may deregulate numerous pathways resulting in the multiple different health outcomes including neurotoxicity, which has been waived from inclusion in the most recent human health risk assessments [16]. Recently, also the neurotoxicity induced by CYP exposure has attracted increasing attention. Indeed, in mice CYP can pass the blood–brain barrier causing motor and cognitive deficits affecting the hippocampus [17]. However, to date, the mechanism of action for these EDCs remains not fully understood.

Recently, new approaches have been developed to evaluate different mechanisms by which pesticides could impact human health, altering gene regulation. Among these new approaches, epigenetics may be considered as a promising tool. Each organism has a specific epigenetic signature that is partially inherited and partially the result of the uterine life and continues to be modified throughout the entire life. Epigenetics consists in different alterations at DNA, RNA, or chromatin leading to changes in gene transcription that can be transient or persistent. Epigenetic alterations may be a consequence of environmental exposures that in turn interact with the genotype to produce changes in gene expression [18]. MicroRNAs (miRNAs), single-stranded, non-coding RNA molecules of 19–24 nucleotides, have a crucial role in epigenetic alterations because they are responsible for fine adjustments in mRNAs abundance and translation ratio, regulating gene expression through actions that influence several biological programs [19]. MiRNAs play a key role in numerous biological processes, including development, cell proliferation, differentiation, and apoptosis [20,21,22]. Their interactions with environmental toxicants are being studied due to their rising importance as quoted by Lema and Cunningham [23]: “Increasing evidence that the expression of miRNAs is affected by several known toxicants as well as oxidative and other forms of cellular stress certainly suggest an important role of miRNAs in toxicology, which could provide a link between environmental influences and gene expression”. Wirbisky et al. demonstrated that in zebrafish ATZ exposure altered the miRNA levels that participate in various functions including angiogenesis [24]. Interestingly, Huang et al. proved that miRNAs could be a molecular bridge between CYP and macrophage polarization, which led to lung cancer metastasis [25]. Moreover, gestational VNZ exposure could increase miR-132 and miR-195a in penises and testes of the offspring, which may ultimately lead to penile and testicular damage and malformations in the offspring [26]. Even with the deepening of experimental research, many of the complex mechanisms behind miRNA regulation and their role in toxicity still remains largely unexplored.

Therefore, the aims of the study were to identify differentially expressed miRNAs in SH-SY5Y cells exposed to subtoxic concentrations of ATZ, CYP, and VNZ and to conduct a bioinformatics analysis to determine the potential functions of the differentially expressed miRNAs and possible pathways mediating their CNS toxicity.

## 2. Results

### 2.1. ATZ, CYP and VNZ at Subtoxic Concentrations Have No Significant Cytotoxicity to SH-SY5Y Cells

**Figure 2:**
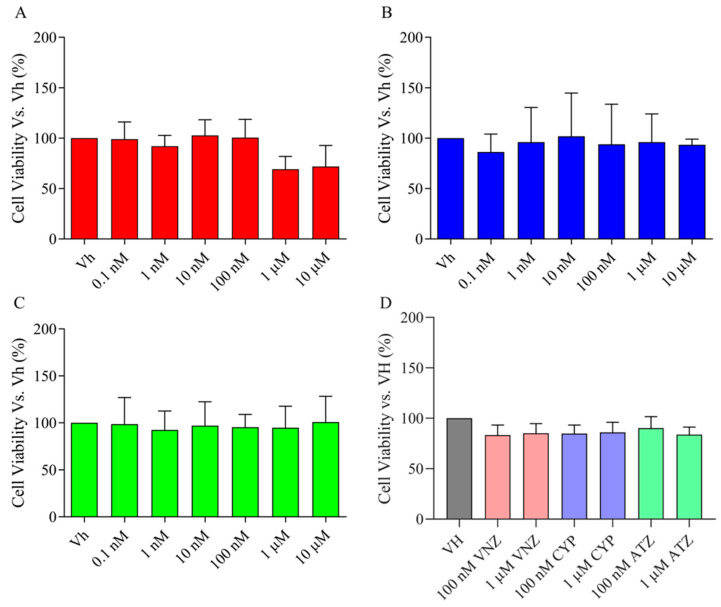
Neuronal viability in undifferentiated and differentiated SH-SY5Y after the treatment with EDCs. Cells were used as undifferentiated (**A**–**C**) or differentiated with retinoic acid (**D**) and treated for 48 h with EDCs. Undifferentiated SH-SY5Y cells were treated with VNZ (**A**), CYP (**B**), or ATZ (**C**) [0.1 nM–10 µM] and cell viability was evaluated by the reduction in MTT to the insoluble formazan. Differentiated SH-SY5Y cells were treated with EDCs 100 nM or 1 μM (**D**). Cell viability was evaluated using the AlamarBlue HSTM test by the conversion of resazurin to resorufin. Data are expressed as fold increases of the percentage of cell viability versus the vehicle group and reported as mean ± SD of three independent experiments (One-way ANOVA, post hoc test Dunnet).

To assess the toxicity of ATR, CYP, and VNZ to undifferentiated SH-SY5Y cells, MTT assays were conducted [27]. As shown in Figure 2A–C, exposure of SH-SY5Y cells to 0.1 nM–10 μΜ ATZ, CYP and VNZ for 48 h did not affect the cell viability. These data indicated that the three EDCs have no significant cytotoxicity to undifferentiated SH-SY5Y cells. Based on these results, EDCs were tested at 100 nM and 1 μΜ in the subsequent experiments. SH-SY5Y cells were induced to differentiate into mature neurons and the AlamarBlue HSTM test was used to assess cytotoxicity [28]. As revealed by the percentage of viability, the EDCs were not cytotoxic also in differentiated SH-SY5Y (Figure 2D).

### 2.2. ATZ, CYP and VNZ at Subtoxic Concentrations Have No Significant Effects on Reactive Oxygen Species Formation

**Figure 3:**
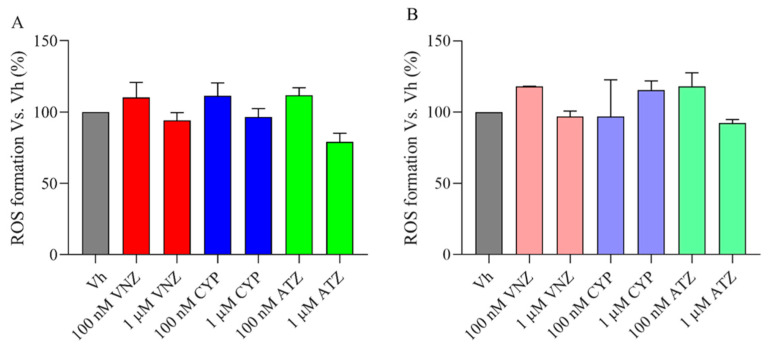
ROS formation in undifferentiated and differentiated SH-SY5Y after the treatment with EDCs. Undifferentiated (A) and differentiated (B) SH-SY5Y cells were treated for 48 h with VNZ, CYP, or ATZ at 100 nM or 1 μM and ROS formation was determined using the fluorescent probe H2DCF-DA. Data are expressed as fold increases in the percentage of ROS formation versus the vehicle group and reported as mean ± SD of three independent experiments (One-way ANOVA, post hoc test Dunnet).

The probe 2′,7′-dichlorodihydrofluorescein diacetate (H_2_DCF-DA) was used to assess the reactive oxygen species (ROS) level in the SH-SY5Y cells [29]. As revealed by the relative percentage of the fluorescence intensity of DCF, the ROS level was not significantly increased by ATZ, CYP, and VNZ 48 h treatment [100 nM, 1 μΜ] in both the undifferentiated (Figure 3A) and differentiated (Figure 3B) SH-SY5Y cells. These data indicated that, at the selected concentrations, ATZ, CYP, and VNZ did not affect cell viability and oxidative stress in SH-SY5Y cells.

### 2.3. Exposure to ATZ, CYP and VNZ at Subtoxic Concentrations Induce Alterations of miRNAs Profile

Using TaqMan array Advanced miRNA (Thermo Fisher Scientific, Waltham, MA, USA), the differential expression of miRNAs in the SH-SY5Y cells from [1 μΜ]-exposed of EDCs and VH were profiled (data not shown). The candidate miRNAs were then selected owing to their reported or predicted role in neuronal proliferation and survival [30,31,32,33,34]. Among them, miR-18b-5p, miR-29b-3p, miR-146b-5p, miR-452-5p, and miR-653-5p were carefully chosen for qRT-PCR validation both in the undifferentiated and differentiated SH-SY5Y cells treated with ATZ, CYP, and VNZ [100 nM, 1 μΜ] for 48 h. The expression levels of miR-18b-5p, miR-29b-3p, miR-146b-5p, miR-452-5p and miR-653-5p were significantly downregulated by EDC treatment compared with the VH (Table 1) in the differentiated SH-SY5Y cells. In particular, ATZ [100 nM] significantly downregulated the expression of miR-18b-5p, miR-29b-3p, miR-146b-5p and miR-653-5p, while VNZ [100 nM] significantly downregulated miR-18b-5p, miR-29b-3p and miR-653-5p and at [1 μΜ] miR-18b-5p, miR-452-5p and miR-653-5p.

**Figure 4:**
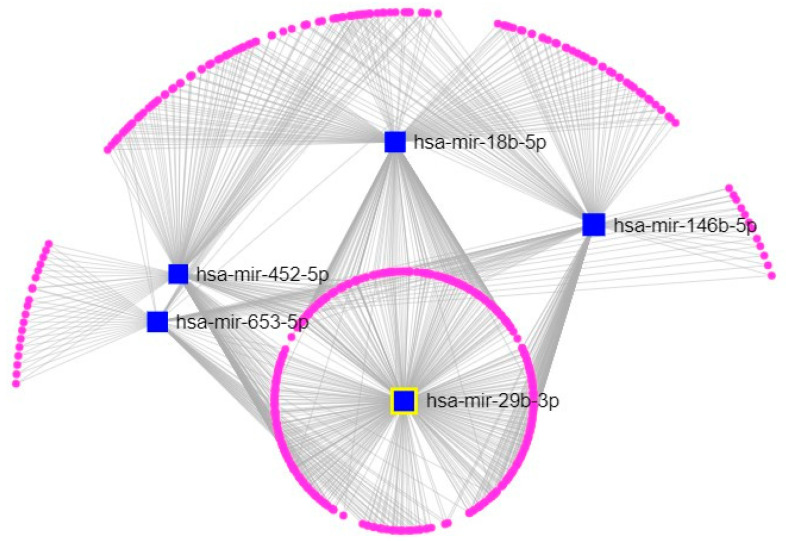
Network gene analysis of the differentially expressed miRNA. The network visualization of the differentially expressed miRNAs and their respective gene targets; blue squares represent miRNAs; purple circles represent genes targets. The visualization is based on significant deregulated miRNAs and their targets of the miRNet platform (https://www.mirnet.ca/) (accessed on 10 September 2022).

Accordingly, to understand the interconnections between the miRNAs identified and their targets, miRNet, a software designed to combine the latest releases from major miRNA annotation databases including miRbase [35] and miRTarBase [36], was used. In the miRNA-target interaction networks, each node represented the gene/miRNA and the edges characterized the connection between nodes. The results obtained by this analysis were able to identify miR-29b-3p as the most connected node to all other miRNAs of interest and genes (Figure 4). Targets were selected by degree filter “all but miRNA nodes”, with a cutoff of 1. For this reason, and because of its involvement in brain function and pathology, miR-29b-3p was chosen for the following analysis [37,38,39,40].

### 2.4. In Silico Prediction of miR-29b-3p Target Genes

**Figure 5:**
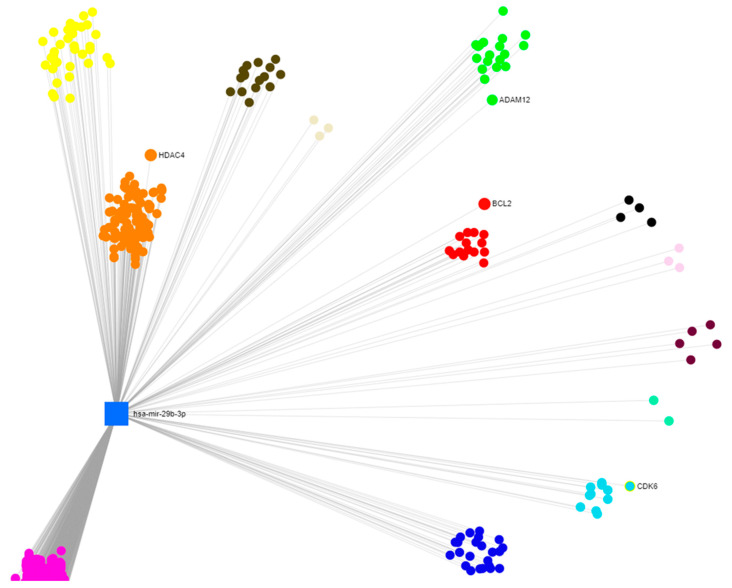
Enrichment analysis for miR-29b-3p obtained from miRNet. The central node is miR-29b-3p, connected to his target genes, grouped according to their integration in one (or more than one) functional pathway identified by the various software. Blue nodes: *pathway in cancer* (KEGG software); green nodes: *signaling by EGFR* (Reactome software); orange: *regulation of cell differentiation*; yellow: *negative regulation of apoptotic processing* (GO:BP software); light blue nodes: *pathway in cancer* and *regulation of cell differentiation*; red nodes: *pathway in cancer, regulation of cell differentiation* and *negative regulation of apoptotic processing*; purple nodes: *pathway in cancer* and *negative regulation of apoptotic processing*; black nodes: *pathway in cancer, signaling by EGFR, regulation of cell differentiation* and *negative regulation of apoptotic processing*; darker green nodes: *pathway in cancer, signaling by EGFR* and *regulation of cell differentiation*; light pink nodes: *pathway in cancer, signaling by EGFR* and *negative regulation of apoptotic processing*; beige: *signaling by EGFR* and *regulation of cell differentiation*; brown nodes: *regulation of cell differentiation* and *negative regulation of apoptotic processing*.

After identifying miR-29b-3p as the miRNA of interest, the study focused on its target genes. To elucidate miRNA functions, the web-based platform miRNet was employed. A total of 1363 target genes targeted by miR-29b-3p were predicted based on them. The network analysis permits to identify the interaction between miRNAs and target genes, and thanks to an enrichment analysis based on the hypergeometric tests, a list of the biological processes associated with the genes targeted by the miRNA of interest was obtained. Initially, using the platform miRNet, the genes whose expression is modulated by the activity of miR-29b-3p were determined; this analysis was able to identify both physiological and pathological pathways in which the genes at issue are involved, drawing information from different databases. Subsequently, it was necessary to select only the functions of interest for the study, namely the pathways that can be modulated by the exposure to EDCs (Figure 5), considering a *p*-value < 0.001 and also the number of gene target.

Particularly, for the KEGG software, the function “pathway in cancer”, which presents a *p*-value of 2.83 × 10^−14^, was selected as the exposure to EDCs may be associated to multiple adverse health outcomes, including cancer [41]. For Reactome, it was selected the function “signaling by epidermal growth factor receptor (EGFR)”, with a *p*-value of 2.4 × 10^−5^, since it has been previously reported that EDCs can directly induce its inhibition [42]. Finally for GO:BP it was chosen “regulation of cell differentiation”, with a *p*-value of 9.7 × 10^−6^, and also “negative regulation of apoptotic processing”, *p*-value 8.1 × 10^−4^, two fundamental functions that can be deregulated following exposure to EDCs [43,44,45]. The genes involved in the four pathways selected are summarized in Table S1. This first screening was able to identify a pool of genes, Bcl2, CDK6, ADAM12 and HDAC4, present in at least one single function. In particular, CDK6 was chosen for the function “pathway in cancer”, ADAM12 for the function “signaling by EGFR”, HDAC4 was selected for the function “regulation of cell differentiation”, and finally Bcl2 for the function “negative regulation of apoptotic processing”. It should also be considered that CDK6 is also present in the “regulation of cell differentiation” pathway, as long as Bcl2 is included not only in “negative regulation of apoptotic processing” but also in “regulation of cell differentiation” and “pathway in cancer”.

### 2.5. Subtoxic Concentrations of VNZ Downregulate miR-29b-3p Contributing to the Increased Level of ADAM12 and CDK6 Genes in Differentiated SH-SY5Y Cells

The total RNA, extracted from the differentiated SH-SY5Y cells and treated with VNZ, CYP, and ATZ at the concentration of 100 nM or 1 µM for 48 h, was reverse transcribed using the High-Capacity RNA-to-cDNA kit. Relative quantification was conducted employing the PowerUp SYBR Green Master Mix assay and the qRT-PCR, with GAPDH as the endogenous control. The analysis showed a significant upregulation, compared to the VH, of the genes ADAM12 and CDK6 after the cells were exposed to VNZ at both concentrations (Table 2).

### 2.6. Subtoxic Concentrations of VNZ Downregulate miR-29b-3p Contributing to the Activation of PI3K/Akt/mTOR Pathway and Suppress p53 Activation in Differentiated SH-SY5Y Cells

**Figure 6:**
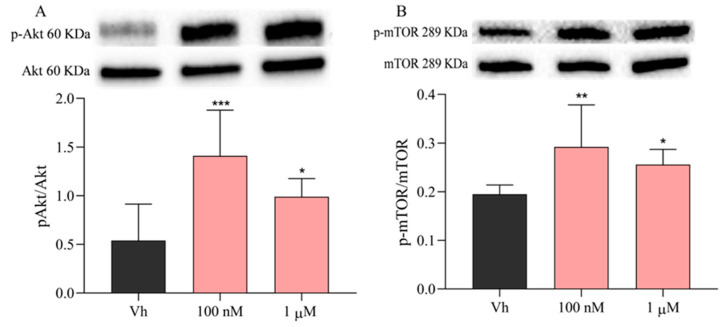
Phoshporilation of Akt and mTOR in differentiated SH-SY5Y cells after the treatment with VNZ. Cells were differentiated with retinoic acid and then treated for 48 h with VNZ 100 nM or 1 μM. The phosphorilation of Akt (**A**) and mTOR (**B**) was evaluated by Western Blotting. Data are expressed as the ratio between the phosphorylated form and the total protein expression and reported as mean ± SD of three independent experiments ((**A**): * *p* < 0.05 and *** *p* < 0.001 vs. VH; (**B**): * *p* < 0.05 and ** *p* < 0.01 vs. VH. One-way ANOVA, post hoc test Dunnett).

Because it has been demonstrated that ADAM12 mediates cell proliferation through the activation of the PI3K/Akt/mTOR pathway, which is involved in several cellular functions and often contributes to oncogenesis [46,47], the phosphorylation levels of Akt (Figure 6A) and mTOR (Figure 6B) were investigated [48]. As shown in Figure 6A, the treatment of 48 h with both concentrations of VNZ (100 nM or 1 µM) induced a significant increase in phospho-Akt (p-Akt) as compared to the VH group. In addition, also the phosphorylation of mTOR was significantly increased by the treatment with VNZ at both concentrations (Figure 6B).

**Figure 7:**
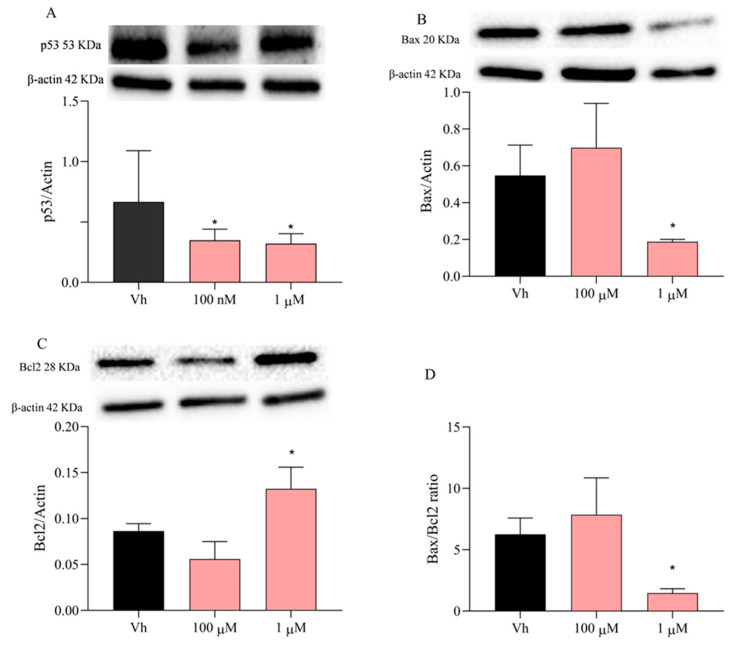
Levels of p53, Bax, and Bcl2 in differentiated SH-SY5Y cells after the treatment with VNZ. Cells were differentiated with retinoic acid and then treated for 48 h with VNZ 100 nM or 1 μM. The levels of p53 (**A**), Bax (**B**) and Bcl2 (**C**) were evaluated by Western Blotting. The ratio between Bax and Bcl2 is represented in panel (**D**). Data are expressed as the ratio between the protein of interest and β-actin expression and reported as mean ± SD of three independent experiments ((**A**): * *p* < 0.05 vs. VH; (**B**): * *p* < 0.05 vs. VH; (**C**): * *p* < 0.05 vs. VH; (**D**): * *p* < 0.05 vs. VH. One-way ANOVA, post hoc test Dunnett).

Furthermore, it has been demonstrated that the activation of CDK6 may be responsible for the inhibition of p53 activity, which is observed in the development of most human cancers [49,50]. For this reason, the p53 levels were assessed and results show that the treatment of differentiated SH-SY5Y cells for 48 h with VNZ at both concentrations reduced them significantly (Figure 7). Although the gene expression of Bcl2 showed a not significant upregulation, the modulation of Bax and Bcl2 was investigated through Western blotting at the same time and concentrations. The protein levels of Bax decreased significantly after 48 h treatment with 1 µM VNZ (Figure 7B); moreover, Bcl2 increased significantly with 1 µM treatment of VNZ (Figure 7C). These results suggested that the decreased in p53 leads to a decrease in the Bax/Bcl2 ratio (Figure 7D) that could alter the cell hemostasis and apoptosis regulation.

## 3. Discussion

In 2013, the World Health Organization (WHO) identified a list of 177 EDCs known to have an impact on the CNS [51]. Unfortunately, the brain is highly susceptible to EDCs, which can cause widespread disruption of hormone receptors, enzymes, and nerve signals. Moreover, the maintenance, renewal, and death of neurons are highly hormone-sensitive. The exposure to EDCs has been extensively studied in relation to human reproduction, while their effects on the CNS are not well understood yet. MiRNAs are fine proteostasis regulators and their involvement in neurotoxicity after EDC exposure has recently attracted the interest of researchers [52,53]. MiRNA expression varies spatially and temporally to fine regulate the biological levels of their target molecules, maintaining the expression profile of the transcriptome and the proteome [39]. Furthermore, the involvement of miRNAs in tumorigenesis is already known; in particular, downregulation of miR-29b-3p has been reported in various human cancers such as prostate cancer, lung cancer, breast cancer, and leukemia [54,55,56,57]. Here, we investigated the effects of the exposure of the SH-SY5Y cells to subtoxic concentrations of VNZ, ATZ, and CYP for 48 h. In this view, the EDC concentrations were selected to not affect cell viability and oxidative stress. Firstly, a profiling of miRNA differently expressed after the EDC exposure was performed and then five miRNAs (miR-18b-5p, miR-29b-3p, miR-146b-5p, miR-452-5p and miR-653-5p), known to be involved in neuronal proliferation and survival, were validated. After a first evaluation on the not differentiated SH-SY5Y cells, the effects of EDC exposure were investigated on the differentiated cells, which represent a model more similar to mature neurons [58].

To better understand the interconnection among all miRNAs selected and between genes and miRNAs, a bioinformatic investigation has been performed to recognize miR-29b-3p as the target of interest of the present study. MiR-29b-3p is a promising candidate that could play a significant role in neurodegenerative process and neuronal survival. It is highly expressed in the brain, in particular in neurons, microglia, and astrocytes [59], and its deregulation has been related to neurodegeneration [60]. Moreover, Shin et al. have demonstrated that the upregulation of miR-29b-3p can exert an anti-cancer activity in the brain [61].

In light of this, a first analysis on miRNet was performed that was able to find 1363 genes whose expression was modulated by the activity of the miR-29b-3p. These genes are implicated in uncountable and very diverse pathways, ranging from collagen production (COL1A1 gene) to genes that provide indications to form the enzyme histone acetyltransferase (KATNLB1 gene). Therefore, it was necessary to use the miRNet software to identify and subsequently select genes involved in the pathways commonly modulated by EDC exposure. Using different software, four functions were selected. From the KEGG software was chosen the pathway in cancer, in which Bcl2 and CDK6 are target genes related to the activity of EDCs. Bcl2 is also implicated in the regulation of cell differentiation, found in the GO:BP software together with HDAC4 and CDK6, and also in the negative regulation of apoptotic processing. Finally, the signaling by the EGFR function, selected for the software Reactome, involves the gene ADAM12.

The gene expression of the selected genes was analyzed by RT-PCR to better understand if EDC exposure could increase or decrease it. The results showed that the exposure to VNZ at both subtoxic concentrations (1 µM and 100 nM) for 48 h was able to cause an upregulation of the expression of the genes ADAM12 and CDK6.

ADAM12 is a disintegrin and metalloprotease involved in cell signaling [62]. Indeed, it is able to activate the EGFR, which, in turns, induces several cellular events such as cell proliferation, differentiation, and migration [63]. In the human brain, ADAM12 is involved in the pathophysiology of several disorders, such as brain cancer, stroke, Alzheimer’s disease, and experimental autoimmune encephalomyelitis [64,65,66,67]. Luna et al. identified ADAM12 as a direct target of miR-29; indeed, miR-29b mimic significantly reduced luciferase expression in cells cotransfected with the 3′-untranslated region (UTR) of ADAM12 [68]. In addition, ADAM12 is a direct target gene of miR-29b in human breast cancer cells and miR-29b downregulated ADAM12 expression in rat renal cells [69,70]. In line with these findings, the present study demonstrated that the treatment with VNZ downregulated miR-29b-3p, which induced significantly the expression of ADAM12, and, in turn, may promote cell proliferation through the PI3K/Akt/mTOR pathway, as demonstrated by the increased phosphorylation of both Akt and mTOR in differentiated SH-SY5Y cells.

CDK6, a cyclin-dependent kinase, is a critical regulator of G1/S transition signaling, and its aberrant activation often leads to uncontrolled cell proliferation and consequently cancer development [71]. Moreover, CDK6 predictively contains putative binding sites for miR-29b-3p in the 3′-UTR. Indeed, Ji et al. demonstrated that overexpression of CDK6 in miR-29b-3p-transfected MDA-MB-231 and Hs578t cells attenuated the inhibitory effect of miR-29b-3p on multiple cancer-related functions, including cell growth, cell G1/S transition, and cell migration [72]. Notably, Zhao et al. showed that the expression levels of the miR-29 family are associated with prognosis in mantle cell lymphoma (MCL). They also found that CDK6 is a direct target of miR29 and the downregulation of the miR-29 family results in upregulation of CDK6 in MCL [73]. These findings suggested that the biological effects of miR-29b-3p could be attributable to the altered CDK6 signaling. Our results confirmed in a human neuronal cell line that the downregulation of miR-29b-3p caused an increase in CDK6 expression that could suppresses p53 responses upon oncogenic stress, inducing the transcription of a number of genes, which negatively regulate p53 [74]. Interestingly, the miR-29 family members up-regulate p53 protein levels and induce p53-mediated apoptosis through repression of p85α, a regulatory subunit of PI3K. The PI3K/Akt/mTOR pathway can negatively regulate p53 activity through the direct phosphorylation and activation of MDM2 by Akt [75]. By targeting p85α, the miR-29 family members reduce PI3K/Akt/mTOR activity and this results in the reduced phosphorylation of Akt and MDM2, which in turn leads to the activation of p53 [76]. In this study, the downregulation of miR-29b-3p by VNZ treatment led to an increased activity of PI3K/Akt/mTOR, which could induce the decrease in p53 and its downstream targets, such as Bax and Bcl2. Chen et al. [77] demonstrated that the p53-dependent signal transduction pathway could affect cellular proliferation by exposure to a variety of environmental toxicants. Our findings suggest that, although EDCs may not be a carcinogenic risk, the exposure to subtoxic concentrations, which resulted in suppression of p53, may serve as a predisposing factor in cancer induction by environmental carcinogens. Moreover, Lee et al. [78] suggested that in self-fertilizing fish the Kryptolebias marmoratus p53 gene would be involved in cellular defense mechanism in early stage of exposure to EDCs and long-term exposure may suppress its expression. It may be possible that the suppression of p53 by EDCs may predispose the host to environmental chemical carcinogenesis.

Since miRNAs are essential modulators of numerous physiological processes, variations in their levels or any other form of disruption of their function have important consequences in human diseases. Overall, this study indicates that the exposure at the subtoxic concentration of VNZ can downregulate miR-29b-3p expression leading to the perturbation of the PI3K/Akt/mTOR pathway, which regulates cell proliferation, differentiation, autophagy, and apoptosis. Downregulation of miR-29b-3p mediated by VNZ is able to increase the gene expression of ADAM12 and CDK6, which may promote a pro-oncogenic response through the activation of the PI3K/Akt/mTOR pathway and counteracting p53 activity.

Our results, along with epidemiological studies, drown attention to the danger of VNZ in the CNS even at low doses. Future research investigating the mode of action of these EDCs in more complex models is needed to understand how these chemicals may produce effects that enable carcinogenesis.

## 4. Materials and Methods

### 4.1. Chemicals

ATZ, CYP, VNZ, H_2_DCF-DA, eosin, thiazolyl blue tetrazolium bromide (MTT), retinoic acid, leupeptin, phenylmethylsulfonyl fluoride (PMSF) and β-actin antibody were purchased from Merck Life Science S.r.L. (Milan, Italy). Dulbecco’s Modified Eagle Medium (DMEM), penicillin and streptomycin mixture, glutamine, trypsin–EDTA, fetal bovine serum (FBS), Dulbecco phosphate buffer saline (DPBS), and Hanks’ balanced salt solution (HBSS), p-Akt, total Akt, phospho-mTOR (p-mTOR), mTOR, p53, Bax, and Bcl2 antibodies were purchased from Cell Signaling Inc. (Euroclone S.p.A, Pero, Italy). β-mercaptoethanol, Sodium dodecyl sulfate (SDS), and Tris·HCl were purchased by Sigma-Aldrich (St. Louis, MO, USA). AlamarBlue HSTM, Pure link RNA mini kit, High-Capacity RNA-to-cDNA kit, PowerUp SYBR Green Master Mix assay were purchased from Thermo Fisher Scientific (Waltham, MA, USA,). Mir-X miRNA First-Strand Synthesis and TB green qRT-PCR assay were purchased from Takara (Mountain View, CA, USA). Quick Start™ Bradford 1× Dye Reagent, Clarity Western ECL Substrate were purchased from Bio-Rad Laboratories S.r.l. (Segrate, Italy). Secondary antibodies peroxidase goat anti-mouse and anti-rabbit were purchased from Jackson ImmunoResearch Europe Ltd. (Cambridgeshire, UK). All of the chemicals used in this study were of the highest purity commercially available.

### 4.2. Cell Culture

#### 4.2.1. SH-SY5Y

Human neuronal SH-SY5Y cells were purchased from the Lombardy and Emilia Romagna Experimental Zootechnic Institute (Brescia, Italy). SH-SY5Y cell line was routinely grown at 37 °C in a humidified incubator with 5% carbon dioxide (CO_2_) in DMEM without phenol red supplemented with 10% FBS, 2 mM glutamine, 50 U/mL penicillin, and 50 μg/mL streptomycin.

#### 4.2.2. SH-SY5Y Differentiation

SH-SY5Y cells were seeded in a 6-well plate at 6 × 10^4^ cells/well in DMEM without phenol red supplemented with 10% FBS. After 5 h, the medium was discarded, and the cells were treated with retinoic acid (10 µM) in DMEM without phenol red supplemented with 2% FBS every 48 h. After 48 h from the third treatment with retinoic acid, cells were treated with EDCs.

### 4.3. EDCs Treatment

To evaluate the neurotoxic effects of VNZ, CYP and ATZ, SH-SY5Y cells were treated with different concentrations of EDCs (0.1 nM, 1 nM, 10 nM, 100 nM, 1 µM, 10 µM) and differentiated cells with 100 nM and 1 µM for 48 h in DMEM without phenol red supplemented with 2% FBS at 37 °C in 5% CO_2_. To obtain the desired concentrations of EDCs, the stock solutions were diluted in complete medium in a maximum of 0.1% DMSO.

### 4.4. Determination of Neuronal Viability

The neuronal viability was assessed by reducing MTT to its insoluble formazan, as previously described [27]. Briefly, SH-SY5Y cells were seeded in a 96-well plate at 1 × 10^4^ cells/well, incubated for 24 h and subsequently treated with different concentrations of VNZ, CYP and ATZ (0.1 nM–10 µM) for 48 h at 37 °C in 5% CO_2_. The treatment medium was then replaced with MTT in HBSS (0.5 mg/mL) for 2 h at 37 °C in 5% CO_2_. Subsequently, washing with HBSS, formazan crystals were solubilized in isopropanol. The amount of formazan was determined using a multilabel plate reader (GENios, TECAN^®^, Mannedorf, Switzerland) at 570 nm and reference filter at 690 nm. Data are expressed as the mean ± SD of viability percentage relative to cells treated with the vehicle (DMSO < 0.1%).

As the differentiated SH-SY5Y cells detached more easily, the neuronal viability was evaluated using the AlamarBlue HSTM test by the conversion of resazurin to resorufin [28]. Briefly, differentiated SH-SY5Y cells were cultured in a 96 well plate at 2 × 10^3^ cells/well and treated with VNZ, CYP and ATZ at the concentration of 100 nM or 1 µM for 48 h at 37 °C in 5% CO_2_. After the treatment, 10 µL of the 10× cell viability reagent was added in each well for 1 h at 37 °C in 5% CO_2_. The amount of resorufin was determined using a multilabel plate reader (GENios) at 570 nm and reference filter at 690 nm. Data are expressed as the mean ± SD of viability percentage relative to cells treated with the vehicle (DMSO < 0.1%).

### 4.5. Determination of ROS Formation Induced by EDCs

The neuronal redox status was evaluated in terms of intracellular ROS levels as previously described [29]. Undifferentiated SH-SY5Y cells were seeded in a 96-well plate at 2 × 10^4^ while differentiated SH-SY5Y cells were seeded in a 6-well plate 6 × 10^4^ cells/well, and subsequently treated with EDCs (100 nM or 1 µM) for 48 h at 37 °C in 5% CO_2_. After the incubation, treatment was removed and 100 µL and 2 mL (for the 96-well plate and the 6-well plate respectively) of the probe H2DCF-DA (10 μg/mL) were added to each well. After 30 min of incubation, the probe was discarded and the intracellular ROS levels were measured (excitation at 485 nm and emission at 535 nm) using a multilabel plate reader (GENios). Data are expressed as the mean ± SD of ROS formation percentage relative to cells treated with the vehicle (DMSO < 0.1%).

### 4.6. RNA Extraction

Total RNA was extracted using Pure link RNA mini kit assay (Thermo Fisher Scientific), as previously described [48]. Briefly, cells pellet was lysate on ice in a lysis buffer with 1% β-mercaptoethanol, added to 70% ethanol and mixed. The solution was filtered in a specific cartridge provided along with the kit assay and washed. RNA was eluted with RNase-free water and quantified by spectrophotometric analysis using NanoDropTM (Thermo Fisher Scientific). Finally, RNA was stored at −80 °C.

### 4.7. Quantitative Real-Time PCR (qRT-PCR) Analysis of miRNA Expression

RNA was reverse transcribed to cDNA for miRNA by Mir-X miRNA First-Strand Synthesis and TB Green qRT-PCR assay (Takara) following the manufacturer’s instructions, and cDNA was stored at −20 °C. MiRNA expression levels were evaluated with the same assay, using miR-U6 as internal reference. The analysis was conducted in samples of SH-SY5Y cells, both differentiated and not differentiated, treated with VNZ, CYP and ATZ at the concentration of 100 nM or 1 µM for 48 h at 37 °C in 5% CO_2_. MiRNA expression was evaluated according to the protocol and run in a 7900HT Fast PCR system (Applied BiosystemsTM, Thermo Fisher Scientific). Each sample was analyzed in triplicate. Quantitative analysis was performed by the 2^−ΔΔCt^ method and vehicle samples were considered as the calibrator of the experiment. The primer sequences are displayed in Table S2. The primers sequence for miRNA validation and gene expression used for qPCR have been drawn using the genome browser Ensembl (Cunningham et al., 2022) and the platform for biotechnology research and development Benchling.

### 4.8. Gene Expression Analysis by RNA Reverse Transcription and qRT-PCR

For each sample, total RNA was reverse transcribed for gene expression analysis using the High-Capacity RNA-to-cDNA kit (Thermo Fisher Scientific), according to the manufacturer’s recommendations, and cDNA was stored at −20 °C. Afterward, relative quantification using PowerUp SYBR Green Master Mix assay (Thermo Fisher Scientific) was performed by qRT-PCR for the genes CDK6, ADAM12, Bcl2, HDAC4-1, HDAC4-2 and GAPDH as endogenous control (Table S3). The analysis was conducted in samples of SH-SY5Y cells differentiated and treated with VNZ, CYP and ATZ at the concentration of 100 nM or 1 µM for 48 h at 37 °C in 5% CO_2_. Each measurement was performed in triplicate and data were analyzed through the 2^−ΔΔCt^ method. Vehicle samples were considered as the calibrator of the experiment.

### 4.9. Western Blotting

The phosphorylation of Akt and mTOR, and the activation of p53, Bax, and Bcl2 were evaluated by using the Western blotting method as previously described [79]. SH-SY5Y cells were seeded in a 6-well plate at 6 × 10^4^ cells/well, differentiated with retinoic acid as described in Section 4.2.2 and subsequently treated with VNZ (100 nM and 1µm) for 48 h at 37 °C in 5% CO_2_.

After the treatment, cells were collected and resuspended in complete lysis buffer containing leupeptin (2 µg/mL), PMSF (100 µg/mL) and a cocktail of protease/phosphatase inhibitors (100×). The protein concentration was assessed using the Bradford method. The protein lysates (30 µg per sample) were then separated by 4–15% SDS polyacrylamide gels (Bio-Rad Laboratories S.r.L.) and transferred onto 0.45 nm nitrocellulose membranes, which were probed with primary antibody p-Akt, p-mTOR, p53, Bax and Bcl2 (all 1:1000; Cell Signaling Inc.), and secondary antibodies. ECL reagents (Bio-Rad Laboratories S.r.L.) were utilized to detect targeted bands. The same membranes were stripped and then re-probed with Akt, mTOR and β-actin antibodies (all 1:1000, raw images are displayed in Figures S1 and S2). Data were normalized and analyzed by densitometry, using Quantity One software (Bio-Rad Laboratories S.r.L.). Data are expressed as the ratio between phosphorylated form and total protein expression, or the ration between the protein of interest and the β-actin signal.

### 4.10. In Silico Target Analysis

The network analysis to predict the target genes of the differentially expressed miRNAs and their over-representation analysis were performed using miRNet [35]. All set of genes targeted by the miRNAs were identified and were used to predict targeted pathways. Functional annotation of the dysregulated miRNA and the identification of miRNA-target gene-controlled pathways were determined via Gene Ontology (GO), Kyoto Encyclopedia of Genes and Genomes (KEGG) and Reactome [80,81,82]. The pathways have been chosen based on the hypergeometric tests with *p*-values ≤ 0.001 and the contribution in the mechanism of the EDCs.

### 4.11. Statistical Analysis

Data were analyzed with the PRISM 9 software (GraphPad Software, La Jolla, CA, USA). The difference between groups was analyzed using one-way ANOVA with Dunnett post hoc test. A difference was considered statistically significant when a *p*-value was less than 0.05.

## 5. Conclusions

In the past few years, several studies have investigated the mechanism by which EDCs act on the different endocrine axis, but there are still many aspects to be clarified. Indeed, to date very few studies have considered the role of miRNA related to endocrine disruption. Although more studies need to be addressed to better understand the effects of EDCs on CNS, the results of the present study show that exposure to low concentration of EDCs may induce different cellular response. Defining the molecular mechanisms behind miRNA changes and the cellular consequences induced by EDCs may contribute to strengthening knowledge about the emerging roles of miRNAs in human health and disease. In conclusion, the comprehension of mechanisms would not only support the causality between EDC exposure and epigenetic changes but also simplify the development of new methods to screen chemicals for their potential to disturb epigenetic patterns.

## Figures and Tables

**Table 1:** qRT-PCR validation of miRNA expression in undifferentiated and differentiated SH-SY5Y cells. Quantitative analysis was performed by the 2^−ΔΔCt^ method and vehicle samples were considered as the calibrator of the experiment.

Undifferentiated Cells						
Target Name	ATZ 100 nM	ATZ 1 µM	CYP 100 nM	CYP 1 µM	VNZ 100 nM	VNZ 1 µM
miR-18b-5p	1.88	1.26	1.28	1.13	1.70	1.74
miR-29b-3p	1.21	0.63	1.53	0.95	1.49	0.78
miR-146b-5p	1.28	1.22	1.47	1.18	1.99	0.93
miR-452-5p	1.94	1.24	1.41	1.45	1.46	1.90
miR-653-5p	1.12	0.80	1.17	1.16	0.95	0.91
**Differentiated cells**						
Target name	ATZ 100 nM	ATZ 1 µM	CYP 100 nM	CYP 1 µM	VNZ 100 nM	VNZ 1 µM
miR-18b-5p	0.30 *	0.65	0.95	0.67	0.26 *	0.32 *
miR-29b-3p	0.42 *	0.92	0.65	0.87	0.38 *	0.70
miR-146b-5p	0.49 *	0.56	1.06	1.32	1.51	0.97
miR-452-5p	0.55	1.09	0.81	0.86	0.57	0.25 *
miR-653-5p	0.41 *	0.59	1.15	1.07	0.26 *	0.32 *

* significantly deregulated miRNAs.

**Table 2:** qRT-PCR validation of gene expression in differentiated SH-SY5Y cells, treated with ATZ, CYP, and VNZ. Quantitative analysis was performed by the 2^−ΔΔCt^ method and vehicle samples were considered as the calibrator of the experiment.

	ADAM12	Bcl2	CDK6	HDAC4
ATZ 100 nM	1.20	1.31	1.28	1.12
CYP 100 nM	1.43	1.56	1.98	1.30
VNZ 100 nM	2.11 *	1.99	2.13 *	1.46
ATZ 1 µM	1.38	1.13	1.58	1.32
CYP 1 µM	1.34	1.05	1.74	1.46
VNZ 1 µM	2.58 *	1.60	2.97 *	1.94

* significantly deregulated genes.

## Data Availability

Data are contained within the article or supplementary material.

## Supplementary Materials

The following supporting information can be downloaded at: https://www.mdpi.com/article/10.3390/ijms232314538/s1, Table S1: Genes involved in the pathways of interest; Table S2: Primers sequence for miRNA validation used for qPCR; Table S3: Primers sequence for gene expression used for qPCR; Figure S1: Raw Western Blotting images of Figure 6; Figure S2: Raw Western Blotting images of Figure 7.
