# Supplementary material for: Effects of Subtoxic Concentrations of Atrazine, Cypermethrin, and Vinclozolin on microRNA-Mediated PI3K/Akt/mTOR Signaling in SH-SY5Y Cells
Source: Int J Mol Sci. 2022 Nov 22;23(23):14538. doi: 10.3390/ijms232314538 (PMC9737829; doi:10.3390/ijms232314538)
Supplement: Supplementary file 1 [file ijms-23-14538-s001.zip › ijms-2040931-supplementary.pdf]

Table S1. Genes involved in the pathways of interest

Pathways in cancer		Signaling by EGFR		Regulation of cell differentiation			Negative regulation of apoptotic process	
**KEGG map05200**		**Reactome R-HSA-177929**		**GO:BP GO:0045595**			**GO:BP GO:0043066**	
BCL2	RALA	CDC42	TNRC6A	CASP8	SPAG9	ZFP36	COL2A1	GPI
CASP8	IGF1	EREG	TNRC6B	CDC42	IGF1	YWHAH	GATA3	MAPK8
CDC42	PIAS2	GSK3B	SOS1	CDK6	RUFY3	LIF	GSK3B	DHCR24
CDK6	BAX	MDM2	FOXO3	COL1A1	CNTN1	MAP1B	HMGCR	SET
COL4A1	BIRC5	PDGFA	CDKN1A	EREG	VIM	ANKRD17	ITGA6	SNAI1
COL4A2	ABL1	PDGFB	EPS15L1	GATA3	TIMP2	APC	ITGB1	CDKN1A
COL4A5	HIF1A	PDGFRA	AKT1	GSK3B	USH2A	CAPRIN1	LRP6	GLO1
COL4A6	STK4	PDGFRB	RPS27A	ID3	PRDM1	RC3H1	MCL1	STAT5A
FOS	XIAP	PIK3R1	UBC	IFNG	SPEN	KLF4	MDM2	APC
GSK3B	CCNE1	PTEN	CAMK4	INSIG1	MEF2A	FBN2	MYC	THBS1
ITGA6	MAPK8	ADAM12	SPRY1	ITGB1	TRIB2	BNIP2	NOTCH2	BNIP2
ITGB1	SUFU	AP2B1	RICTOR	LOXL2	SMC1A	ZFHX3	PIK3CG	TP53
LAMA2	MAPK10	PHLPP2	FRS2	LRP6	FNDC3B	TP53	PIK3R1	CBX4
LAMC1	CCND1	PAG1	NRAS	MEN1	PCM1	AKT1	PRKAA1	AKT1
LAMC2	FN1	EPS15	CALM3	MYC	MEF2C	ARNT	PTEN	HSPD1
MDM2	SOS1			MYCN	BAX	NTRK2	TGFB3	NTRK2
MMP2	PIK3R3			NOTCH2	GLG1	TCF7L2	TNFAIP3	TCF7L2
MMP9	CDKN1A			PDGFRA	DPYSL2	CAMK4	TPT1	PGAP2
MYC	STAT5A			PIK3R1	SIRT1	PTPRD	VEGFA	ATM
PDGFA	TP53			PLAG1	ABL1	LRP8	VHL	TSC22D3
PDGFB	AKT1			PPARD	DDX17	SKI	BTG2	SON
PDGFRA	ARNT			MAP2K6	RBFOX2	CHRNB2	HMGA2	ITGA5
PDGFRB	SKP2			PTEN	HIF1A	ARPC2	SH3GLB1	PEA15
PIK3CG	TCF7L2			REST	DICER1	PRKCI	ANGPTL4	PRKCI
PIK3R1	DVL3			S100B	MIB1	ARF6	RHBDD1	ANXA5
PPARD	FZD5			STAT3	CA2	CTNNB1	KDM1A	CTNNB1
PTEN	CTNNB1			DYNLT1	STMN2	HOOK3	IGF1	SERPINB9
STAT3	CYCS			TGFB1	TRPS1	SDC2	RB1CC1	SCG2
TGFB1	RELA			TGFB2	NEFM	ROBO1	YBX3	PDE3A
TGFB2	SMAD2			TGFB3	EZH2	ASXL1	MEF2C	RELA
TGFB3	JUN			TPT1	SUFU	PDE3A	BAX	LIG4
VEGFA	NRAS			VEGFA	DDX5	RELA	BIRC5	JUN
VHL	AKT2			VHL	PMP22	LIG4	G2E3	HTT
AKT3				HMGA2	CCND1	SH3PXD2B	SIRT1	TOPORS
				TCL1A	SMAP1	SMAD2	HIF1A	NUP62
				NCOA3	PPP2CA	LPL	DICER1	NRAS
				NREP	ACVR2B	SOX11	XIAP	DDX3X
				MORF4L2	CCDC88A	ULK1	PIM2	BCL2
				HDAC4	BCL9	JUN		
				TESPA1	ERRFI1	FAM20C		
				CIT	CNR1	MED14		
				WWTR1	MED28	SS18L1		
				ASXL2	FOXO3	BCL9L		
				CTNNBIP1	BCL11A	TCF4		
				TET1	SNAI1	MAFG		
				CD276	RUNX2	LPAR1		
				ZFPM1	PRMT1	MAFB		
				KDM1A	STAT5A	BCL2		

Table S2. Primers sequence for miRNA validation used for qPCR

miRNA	Primer Sequence (5′-3′)	Utilization
miR-18b-5p	Forward: TAAGGTGCATCTAGTGCAGTTAG<br>Reverse: mRQ 3' Primer (TaKaRa)	qPCR
miR-29b-3p	Forward: TAGCACCATTTGAAATCAGTGTT<br>Reverse: mRQ 3' Primer (TaKaRa)	qPCR
miR-146b-5p	Forward: TGAGAACTGAATTCCATAGGCTG<br>Reverse: mRQ 3' Primer (TaKaRa)	qPCR
miR-452-5p	Forward: AACTGTTTGCAGAGGAAACTGA<br>Reverse: mRQ 3' Primer (TaKaRa)	qPCR
miR-653-5p	Forward: GTGTTGAAACAATCTCTACTG<br>Reverse: mRQ 3' Primer (TaKaRa)	qPCR
Mir-U6	Forward: mRQ 5' Primer (TaKaRa)<br>Reverse: mRQ 3' Primer (TaKaRa)	qPCR

Table S3. Primers sequence for gene expression used for qPCR

Gene	Primer Sequence (5′-3′)	Utilization
ADAM12	Forward: GATGTCTCCCTCGCTCGAAA	qPCR
	Reverse: GTCCCCTGAGACCAGAACAC	
	Forward: GTCGGAGGGAGCATGATCAT	
	Reverse: TTGATCTCCACCCGCACAAT	
BCL2	Forward: ATGTGTGTGGAGAGCGTCAA	qPCR
	Reverse: AGTTCCACAAAGGCATCCCA	
CDK6	Forward: TGGATCTCTGGAGTGTTGGC	qPCR
	Reverse: GGGAGTCCAATCACGTCCAA	
HDAC4	Forward: CGACGCCAAAGATGACTTCC	qPCR
	Reverse: CGTCTTTCGGCCACTTTCTG	
	Forward: CAGTACGGACGGGTCACTAC	
	Reverse: ATTGGCTTTGTGTCCTTGGC	

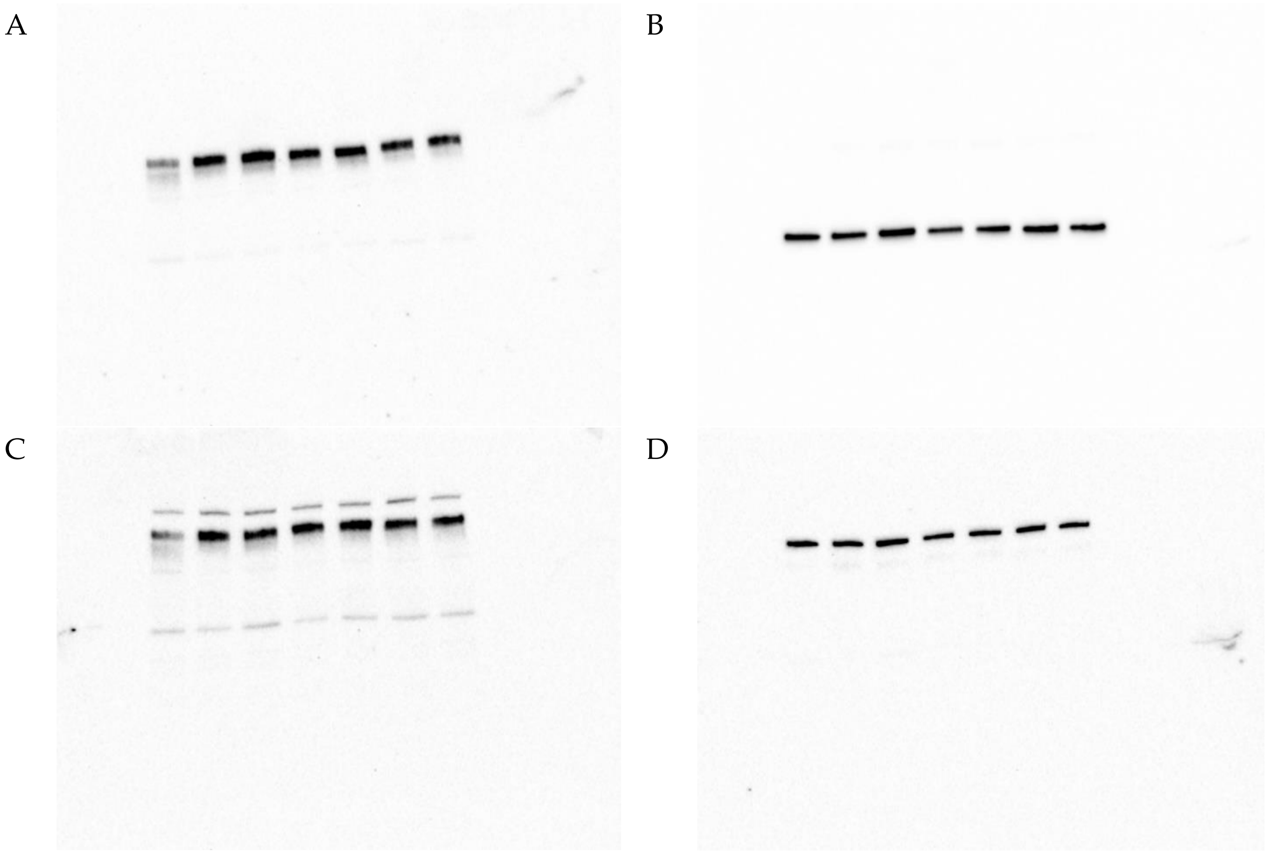

Figure S1. Raw Western Blotting images of Figure 6. Raw images of p-Akt (A), total Akt (B), p-mTOR (C), total mTOR (D).

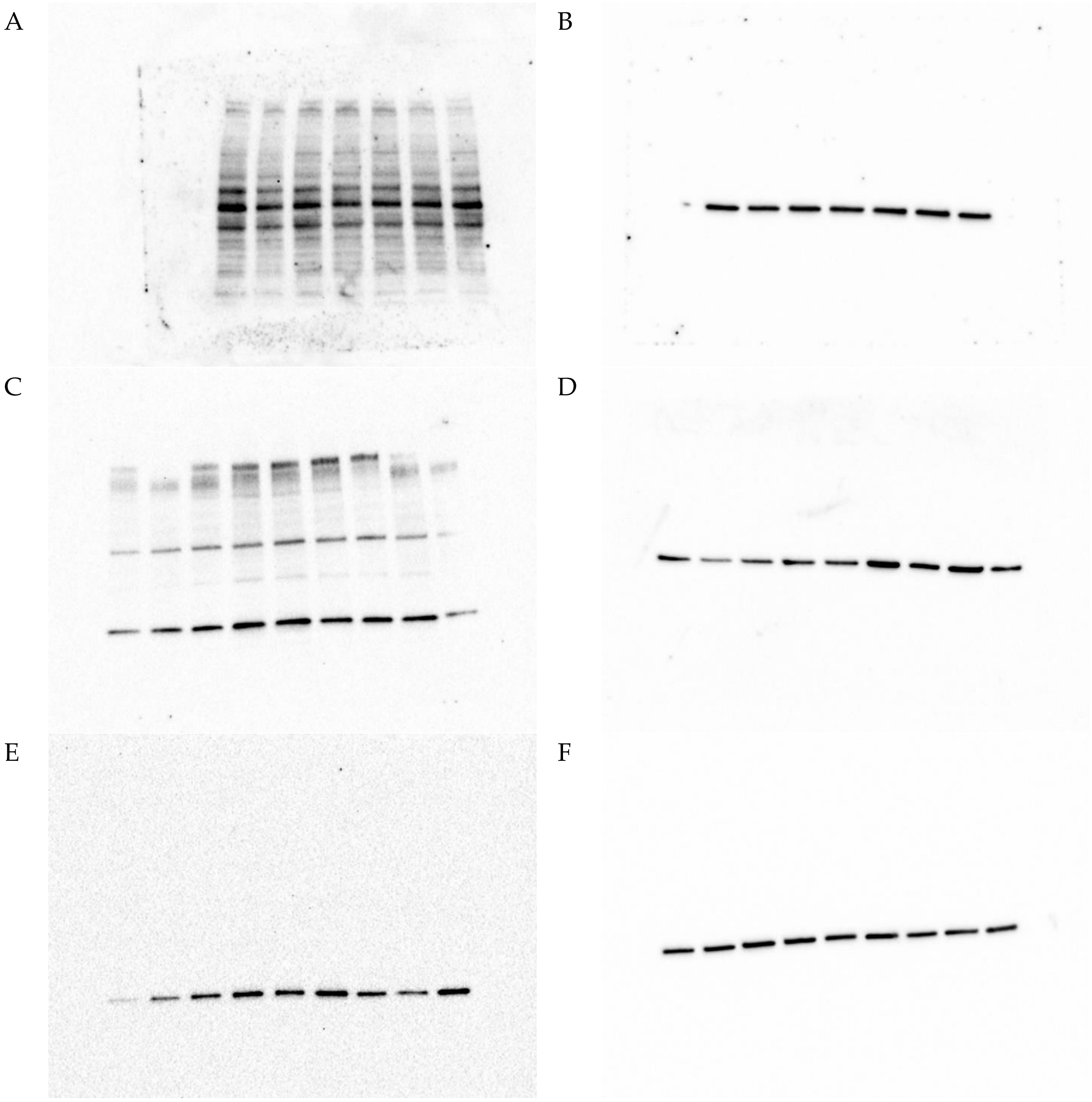

Figure S2. Raw Western Blotting images of Figure 7. Raw images of p53 (A), β-actin 7A (B), Bax (C), β-actin 7B (D), Bcl2 (E), and β-actin 7C (F).
